# Publisher Correction: MethCORR modelling of methylomes from formalin-fixed paraffin-embedded tissue enables characterization and prognostication of colorectal cancer

**DOI:** 10.1038/s41467-020-16538-5

**Published:** 2020-06-03

**Authors:** Trine B. Mattesen, Mads H. Rasmussen, Juan Sandoval, Halit Ongen, Sigrid S. Árnadóttir, Josephine Gladov, Anna Martinez-Cardus, Manuel Castro de Moura, Anders H. Madsen, Søren Laurberg, Emmanouil T. Dermitzakis, Manel Esteller, Claus L. Andersen, Jesper B. Bramsen

**Affiliations:** 1 Department of Molecular Medicine, Aarhus University Hospital, 8200 Aarhus, Denmark; 2 Epigenomic Unit, Health Research Institute La Fe (ISSLaFe), Valencia, Spain; 3 Biomarker and precision medicine Unit, Health Research Institute La Fe (ISSLaFe), Valencia, Spain; 4 Genetic Medicine and Development, University of Geneva Medical School-CMU, 1 Rue Michel-Servet, 1211 Geneva, Switzerland; 5 Badalona Applied Research Group in Oncology (B-ARGO), Germans Trias i Pujol Research Institute (IGTP), Badalona, Barcelona, Catalonia Spain; 6 Medical Oncology Service, Institute Catalan of Oncology (ICO), Badalona, Barcelona, Catalonia Spain; 7 Josep Carreras Leukaemia Research Institute (IJC), Badalona, Barcelona, Catalonia Spain; 8 Department of Surgery, Hospitalsenheden Vest, 7400 Herning, Denmark; 9 Colorectal Surgical Unit, Department of Surgery, Aarhus University Hospital, 8200 Aarhus, Denmark; 10 Josep Carreras Leukaemia Research Institute (IJC), Badalona, Barcelona, Catalonia Spain; 11 Centro de Investigacion Biomedica en Red Cancer (CIBERONC), Madrid, Spain; 12 Institucio Catalana de Recerca i Estudis Avançats (ICREA), Barcelona, Catalonia Spain; 13 Physiological Sciences Department, School of Medicine and Health Sciences, University of Barcelona (UB), Barcelona, Catalonia Spain

**Keywords:** Classification and taxonomy, Prognostic markers, Colorectal cancer

Correction to: *Nature Communications* 10.1038/s41467-020-16000-6, published online 24 April 2020.

The original version of this Article contained an error in Fig. 2d, in which all the numbers appeared as bold instead of either normal or bold type setting to indicate statistical significance of FDR < 0.05. This has been corrected in both the PDF and HTML versions of the Article.

