# Supplementary material for: MethCORR modelling of methylomes from formalin-fixed paraffin-embedded tissue enables characterization and prognostication of colorectal cancer
Source: Nat Commun. 2020 Apr 24;11:2025. doi: 10.1038/s41467-020-16000-6 (PMC7181739; doi:10.1038/s41467-020-16000-6)
Supplement: Supplementary file 1 — Supplementary Information [file 41467_2020_16000_MOESM1_ESM.pdf]

Supplementary Information for

MethCORR Modelling of Methylomes from Formalin-fixed Paraffin-embedded Tissue enables Characterization and Prognostication of Colorectal Cancer

By Mattesen et al.

Supplementary Figure 1

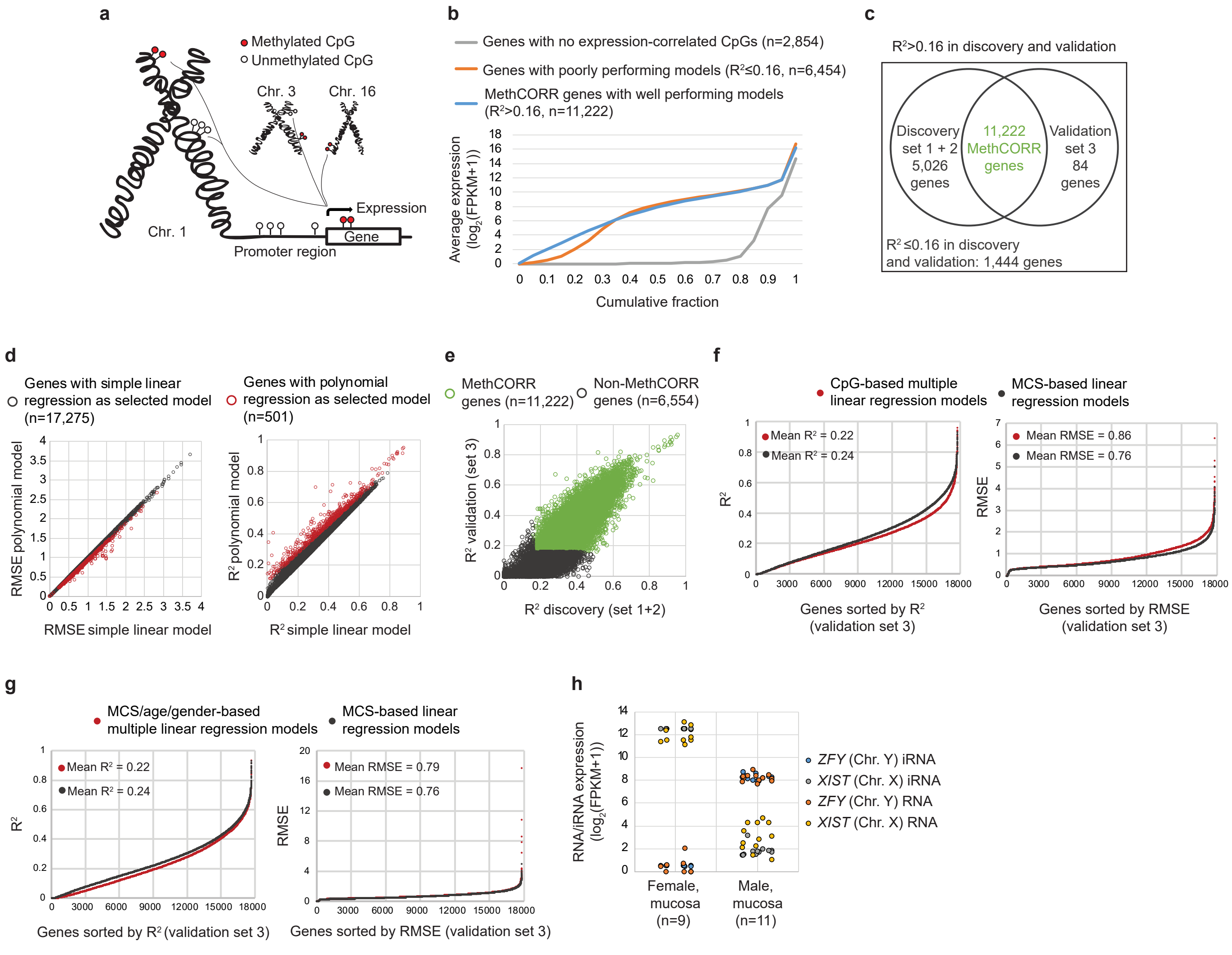

Supplementary Figure 1. Development of the MethCORR approach.

a) Schematic drawing of the MethCORR principle. MethCORR identified RNA expression-correlated CpGs genome-wide for each gene rather than focusing on local gene DNA methylation. Black lines indicate that gene expression can correlate with the DNA methylation status (both hypo- and hyper-methylation) of distantly located CpGs. **b)** Cumulative fraction plot showing the average expression of genes for which no sets of positive and negative expression-correlated CpGs were identified (grey), genes with poorly performing MethCORR models (orange), and MethCORR genes with well performing MethCORR models (blue). Genes with no expression-correlated CpGs are significantly lower expressed than genes with MethCORR models ($P<10^{-100}$, Wilcoxon rank-sum test). **c)** Venn diagram showing the distribution of genes with model performance $R^2>0.16$ in the discovery set 1+2 and the validation set 3. MethCORR genes (n=11,222; shown in green) have $R^2>0.16$ in both discovery and validation sets. **d)** Scatterplot showing the relationship between RMSE (left) or R^2 (right) for simple linear regression gene models and best performing polynomial regression gene models in discovery set 1+2 samples. Genes marked in red exhibit a ≥5% decrease in RMSE as compared to simple linear models and polynomial models were therefore selected for these genes **e)** Scatterplot showing the relationship between R^2 in the discovery set (set 1+2) and validation set 3. MethCORR genes are indicated by green color ($R^2>0.16$) and non-MethCORR genes ($R^2\leq0.16$) are indicated by dark grey color. **f)** Line graphs showing R^2 (left) or RMSE (right) for MCS-based linear regression models (dark grey) or multiple linear regression models using the top ≤200 expression-correlated CpGs for each gene (red). Genes are sorted according to increasing R^2 or RMSE. **g)** Line graphs showing R^2 (left) or RMSE (right) for linear regression models including only MCSs (dark grey) and multiple linear regression models including both MCS, age, and gender (red). Genes are sorted according to increasing R^2 or RMSE. **h)** Scatterplot showing the RNA expression and inferred RNA (iRNA) expression of the gender-specific genes *ZFY* (chromosome Y) and *XIST* (chromosome X) in female and male normal mucosa samples from the COREAD dataset.

Supplementary Figure 2

a

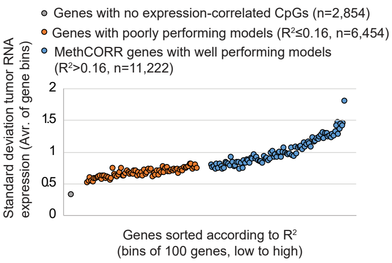

b

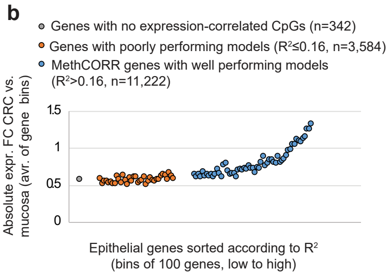

c

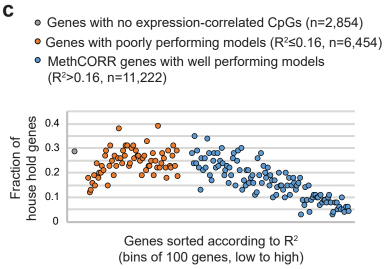

d

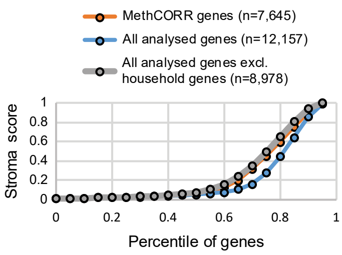

e

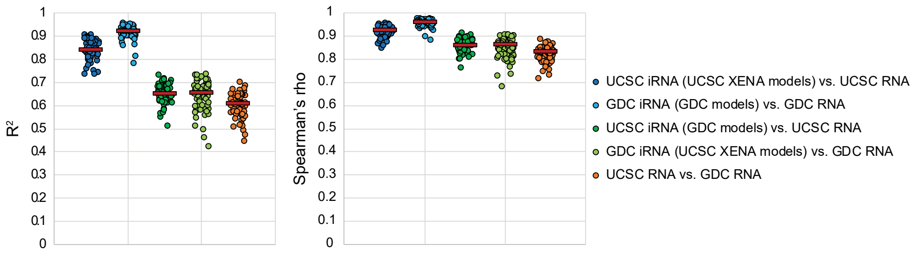

f

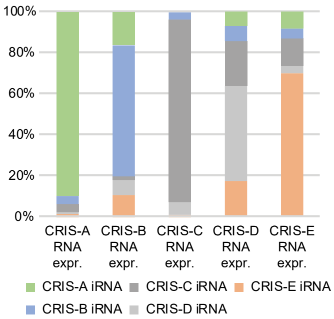

g

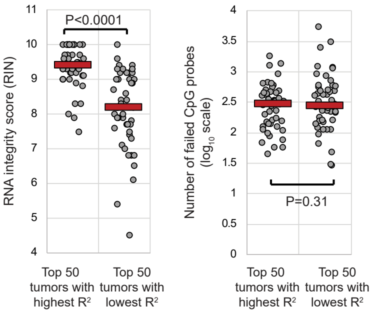

h

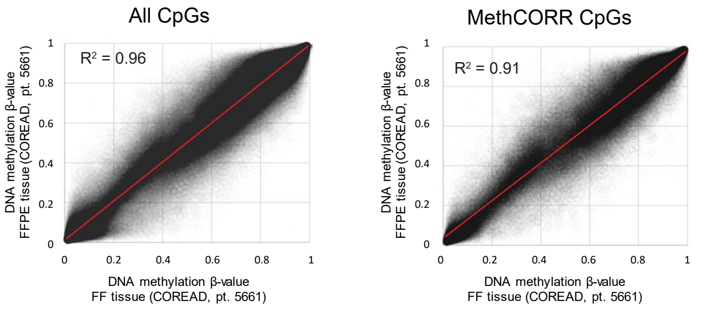

TCGA COREAD patient Id	R^2 for all CpG´s FF vs. FFPE	R^2 for methCORR CpG´s FF vs. FFPE	File Ids (GDC)
Pt. 2677	0.95	0.90	jhu-usc.edu_COAD.HumanMethylation450.11.lvl-3.TCGA-A6-2677-01A-01D-A27A-05.gdc_hg38;<br>jhu-usc.edu_COAD.HumanMethylation450.11.lvl-3.TCGA-A6-2677-01B-02D-A27A-05.gdc_hg38
Pt. 2684	0.95	0.91	jhu-usc.edu_COAD.HumanMethylation450.1.lvl-3.TCGA-A6-2684-01A-01D-1407-05.gdc_hg38;<br>jhu-usc.edu_COAD.HumanMethylation450.1.lvl-3.TCGA-A6-2684-01A-01D-A27A-05.gdc_hg38;<br>jhu-usc.edu_COAD.HumanMethylation450.1.lvl-3.TCGA-A6-2684-01C-08D-A27A-05.gdc_hg38
Pt. 3809	0.94	0.91	jhu-usc.edu_COAD.HumanMethylation450.12.lvl-3.TCGA-A6-3809-01A-01D-A27A-05.gdc_hg38;<br>jhu-usc.edu_COAD.HumanMethylation450.12.lvl-3.TCGA-A6-3809-01B-04D-A27A-05.gdc_hg38
Pt. 3810	0.98	0.96	jhu-usc.edu_COAD.HumanMethylation450.12.lvl-3.TCGA-A6-3810-01A-01D-A27A-05.gdc_hg38;<br>jhu-usc.edu_COAD.HumanMethylation450.12.lvl-3.TCGA-A6-3810-01B-04D-A27A-05.gdc_hg38;
Pt. 5656	0.94	0.90	jhu-usc.edu_COAD.HumanMethylation450.6.lvl-3.TCGA-A6-5656-01A-21D-1837-05.gdc_hg38;<br>jhu-usc.edu_COAD.HumanMethylation450.6.lvl-3.TCGA-A6-5656-01A-21D-A27A-05.gdc_hg38;<br>jhu-usc.edu_COAD.HumanMethylation450.6.lvl-3.TCGA-A6-5656-01B-02D-A27A-05.gdc_hg38
Pt. 5659	0.94	0.91	jhu-usc.edu_COAD.HumanMethylation450.2.lvl-3.TCGA-A6-5659-01A-01D-1651-05.gdc_hg38;<br>jhu-usc.edu_COAD.HumanMethylation450.2.lvl-3.TCGA-A6-5659-01A-01D-A27A-05.gdc_hg38;<br>jhu-usc.edu_COAD.HumanMethylation450.2.lvl-3.TCGA-A6-5659-01B-04D-A27A-05.gdc_hg38
Pt. 5661	0.96	0.91	jhu-usc.edu_COAD.HumanMethylation450.2.lvl-3.TCGA-A6-5661-01A-01D-1651-05.gdc_hg38;<br>jhu-usc.edu_COAD.HumanMethylation450.2.lvl-3.TCGA-A6-5661-01B-05D-2299-05.gdc_hg38
Pt. 5665	0.96	0.92	jhu-usc.edu_COAD.HumanMethylation450.2.lvl-3.TCGA-A6-5665-01A-01D-1651-05.gdc_hg38;<br>jhu-usc.edu_COAD.HumanMethylation450.2.lvl-3.TCGA-A6-5665-01B-03D-2299-05.gdc_hg38
Pt. 6650	0.97	0.98	jhu-usc.edu_COAD.HumanMethylation450.4.lvl-3.TCGA-A6-6650-01A-11D-1772-05.gdc_hg38;<br>jhu-usc.edu_COAD.HumanMethylation450.4.lvl-3.TCGA-A6-6650-01A-11D-A27A-05.gdc_hg38;<br>jhu-usc.edu_COAD.HumanMethylation450.4.lvl-3.TCGA-A6-6650-01B-02D-A27A-05.gdc_hg38
Pt. 6780	0.98	0.93	jhu-usc.edu_COAD.HumanMethylation450.6.lvl-3.TCGA-A6-6780-01A-11D-1837-05.gdc_hg38;<br>jhu-usc.edu_COAD.HumanMethylation450.6.lvl-3.TCGA-A6-6780-01A-11D-A27A-05.gdc_hg38;<br>jhu-usc.edu_COAD.HumanMethylation450.6.lvl-3.TCGA-A6-6780-01B-04D-A27A-05.gdc_hg38
Pt. 6781	0.97	0.89	jhu-usc.edu_COAD.HumanMethylation450.8.lvl-3.TCGA-A6-6781-01A-22D-1926-05.gdc_hg38;<br>jhu-usc.edu_COAD.HumanMethylation450.8.lvl-3.TCGA-A6-6781-01A-22D-A27A-05.gdc_hg38;<br>jhu-usc.edu_COAD.HumanMethylation450.8.lvl-3.TCGA-A6-6781-01B-06D-A27A-05.gdc_hg38

i

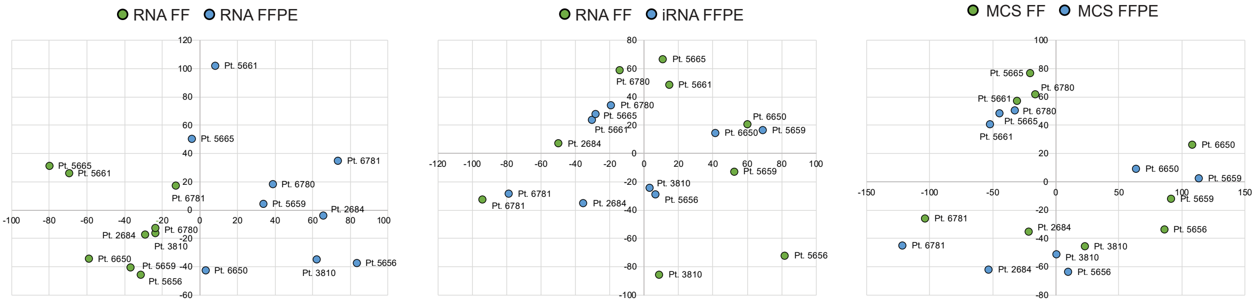

Supplementary Figure 2. Characterization of the MethCORR approach.

a) Graph showing the standard deviation of COREAD RNA expression (bin average) for genes with no expression-correlated CpGs (grey), genes with poorly performing models (orange), and MethCORR genes with good performing models (blue). **b)** Graph showing the absolute gene expression fold-change (FC; bin average) between CRC and normal mucosa samples for epithelial genes with no expression-correlated CpGs (grey), genes with poorly performing models (orange), and MethCORR genes with well performing models (blue). **c)** Graph showing fraction of household genes[1] for genes with no expression-correlated CpGs (grey), genes with poorly performing models (orange), and MethCORR genes with well performing models (blue). **d)** Line graph showing the stroma score[2] distribution, if available, for MethCORR genes (orange), all genes (blue), or all genes excluding household genes (grey). **e)**. Scatterplot showing R^2 (left) or Spearman´s rho (right) for common MethCORR genes and set 3 samples between MethCORR analysis using normalized data acquired either via the UCSC XENA database[3] or NCI genome database commons[4]. **f)** Histogram showing overlap in CRIS subtype[5] predictions for COREAD tumors using RNA or iRNA expression as input. **g)** Scatterplots showing the RNA integrity score (RIN; left) or number of failed CpG probes on the 450K array (detection *P*-value>0.05, ChAMP software[6]; right) for the top 50 SYSCOL tumor samples with the highest and lowest R^2 correlation between RNA and iRNA expression. *P*-values are given (Wilcoxon rank-sum test). **h)** Scatterplots showing the relationship between DNA methylation β-values for a representative COREAD sample with matched fresh-frozen and FFPE tissue for all CpGs included on the HM-450K array (left) or included in the MethCORR matrix (middle). Table (right) with R^2 and file ids for all 11 patients with matched tissue. For six patients, average R^2 values for two fresh-frozen datasets are given. **i)** Scatterplots showing the first principal component and second principal component score from a PCA analysis of nine COREAD samples combining RNA expression in FFPE and matched fresh-frozen tissue (left), iRNA expression calculated in FFPE tissue and RNA expression in fresh-frozen tissue (middle), or MCSs calculated in FFPE tissue and fresh-frozen tissue (right).

Supplementary Figure 3

a

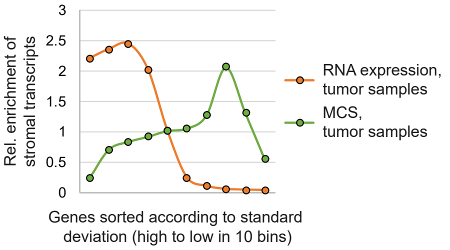

b

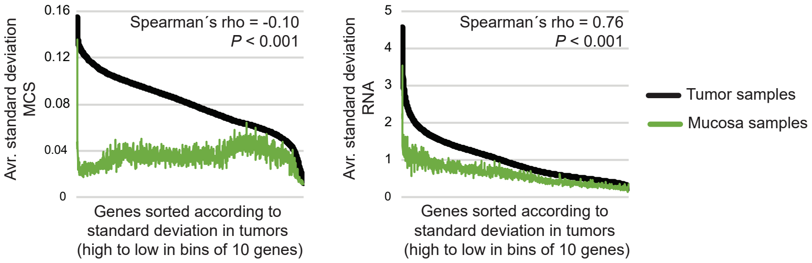

c

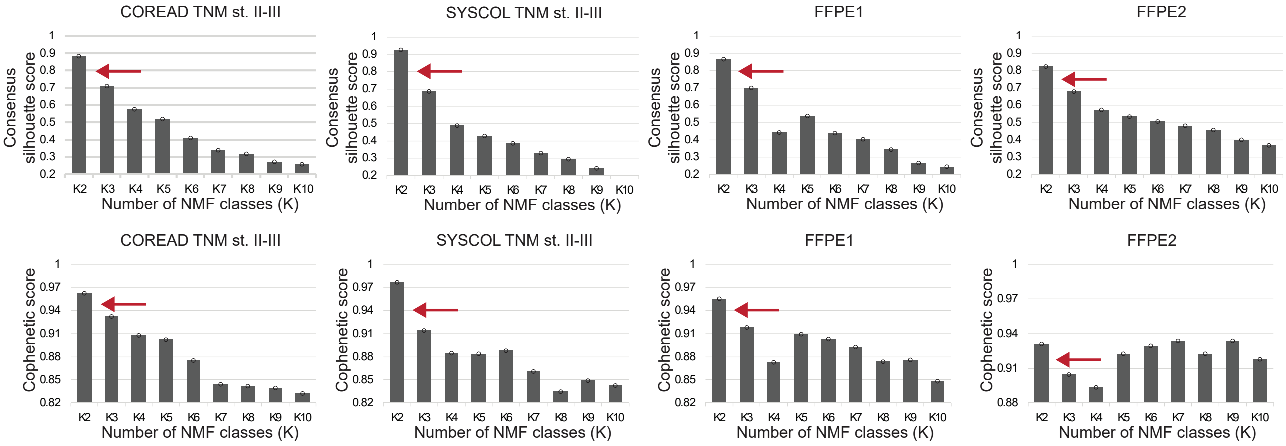

d

FDR *P*-value	COREAD CRC1	COREAD CRC2	SYSCOL CRC1	SYSCOL CRC2	FFPE1 CRC1	FFPE1 CRC2	FFPE2 CRC1	FFPE2 CRC2	MSI/Serrated	Conventional
COREAD CRC1	x	x	0.004	1.000	0.003	1.000	0.003	1.000	0.008	1.000
COREAD CRC2	x	x	1.000	0.003	1.000	0.003	1.000	0.003	1.000	0.004
SYSCOL CRC1	0.004	1.000	x	x	0.003	1.000	0.004	1.000	0.003	1.000
SYSCOL CRC2	1.000	0.003	x	x	1.000	0.003	1.000	0.004	1.000	0.003
FFPE1 CRC1	0.003	1.000	0.003	1.000	x	x	0.005	1.000	0.003	1.000
FFPE1 CRC2	1.000	0.003	1.000	0.003	x	x	1.000	0.005	1.000	0.003
FFPE2 CRC1	0.003	1.000	0.004	1.000	0.005	1.000	x	x	0.016	0.997
FFPE2 CRC2	1.000	0.003	1.000	0.004	1.000	0.005	x	x	1.000	0.027
MSI/Serrated	0.008	1.000	0.003	1.000	0.003	1.000	0.016	1.000	x	x
Conventional	1.000	0.004	1.000	0.003	1.000	0.003	1.000	0.027	x	x

FDR 1 0

e

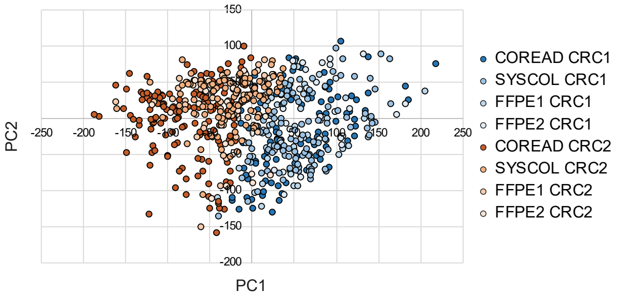

f

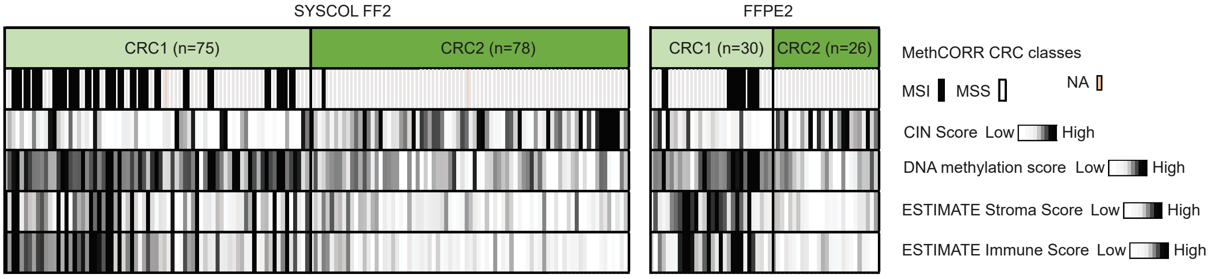

Supplementary Figure 3. MethCORR scores and NMF clustering.

a) Line graph showing the relative enrichment/depletion of stromal transcripts for 10 bins of genes arranged according to decreasing standard deviation in either RNA expression (orange) or MCSs (green) among COREAD tumor samples. MCSs exhibit a relative greater inter-tumor variation for cancer cell-derived transcripts than for stromal transcripts as compared to RNA expression. **b)** Line graphs showing the standard deviation of MCSs (left) and RNA expression (right) within COREAD normal mucosa (green) and tumor samples (black). Genes are sorted according to decreasing standard deviation within tumor samples. Genes with greatest inter-tumor variation in MCSs exhibited little variation in normal mucosa samples, indicating that MCSs exhibit greatest variation for genes deregulated in CRC. In contrast, inter-tumor and inter-mucosa RNA profile variation are positively correlated (Spearman´s Rho=0.76 and *P*-value<0.001), indicating that much of the RNA expression variation is not reflecting CRC-related traits. **c)** Histograms showing consensus silhouette scores (upper panel) and cophenetic scores (lower panel) from consensus NMF[7]-clustering's of COREAD, SYSCOL, FFPE1, and FFPE2 TNM stage II-III samples (Supplementary Table 1). Clustering's were performed for 2 to 10 classes using MCSs as input and the number of classes was chosen according to where the silhouette/cophenetic score first exhibited a prominent reduction (red arrow). **d)** Table showing FDR *P*-values for pair-wise comparative analysis of MethCORR subtypes in the four CRC cohorts and MSI/serrated vs. conventional CRC (from the dataset GSE68060[8]) using the SubMap tool[9]. **e)** Scatterplot showing the first principal component (X-axis) and second principal component score (Y-axis) from a PCA analysis of the COREAD, SYSCOL, FFPE1, and FFPE2 cohorts together using MCSs as input. **f)** Main molecular features of the CRC1 and CRC2 MethCORR subtypes in the SYSCOL FF1 and the FFPE2 cohort (Supplementary Table 1). MSI and MSS status is indicated in black and white whereas unavailable values are orange. CIN scores were derived for SYSCOL FF2 and FFPE2 samples using 450K methylation data and sample DNA methylation scores were calculated as the 40th percentile of DNA methylation β-values for all CpGs. The Stroma- and Immune Scores were generated from MCSs using ESTIMATE[10].

Supplementary Figure 4

a

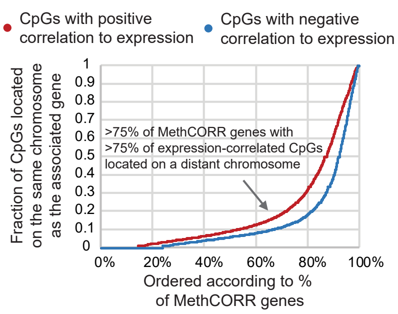

b

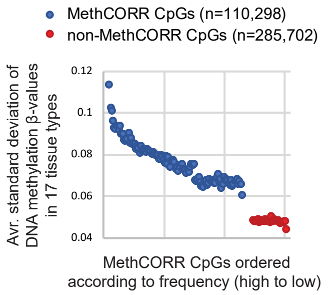

d

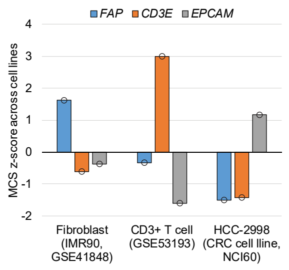

c

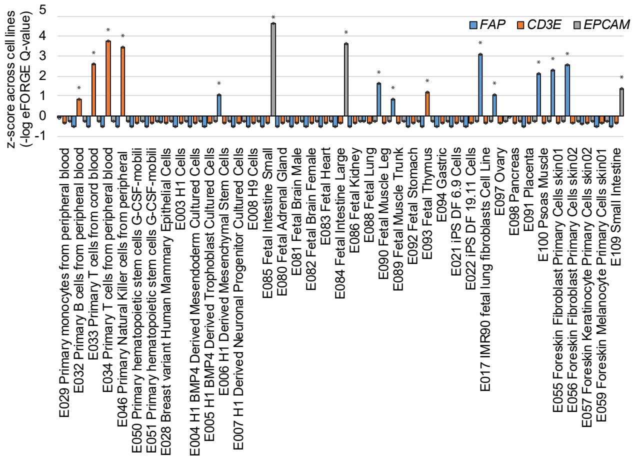

e

TOP 50 genes with high CpG overlap to	Gene set (ARCHS4 Tissues)	Rank in prediction	*P*-value	Adjusted *P*-value
CD3E	CD4+ T CELL	1	8.191E-39	8.847E-37
CD3E	TLYMPHOCYTE	2	5.887E-37	3.179E-35
CD3E	REGULATORY T CELLS	3	3.448E-35	1.241E-33
CD3E	NATURAL KILLER CELLS	4	1.692E-33	4.567E-32
CD3E	BLOOD PBMC	5	2.583E-30	5.579E-29
FAP	OSTEOBLAST	1	2.341E-27	2.528E-25
FAP	ASTROCYTE	2	5.929E-26	3.202E-24
FAP	FIBROBLAST	3	2.78E-23	1.001E-21
FAP	STROMAL CELL	4	2.78E-23	7.506E-22
FAP	VASCULAR SMOOTH MUSCLE	5	5.196E-22	1.122E-20
EPCAM	COLON (BULK TISSUE)	1	3.459E-15	3.736E-13
EPCAM	ILEUM (BULK)	2	3.365E-13	1.817E-11
EPCAM	SMALL INTESTINE (BULK TISSUE)	3	4.714E-08	0.000001697
EPCAM	GASTRIC TISSUE (BULK)	4	0.000006075	0.000164
EPCAM	GASTRIC EPITHELIAL CELL	5	0.0003716	0.008026

Supplementary Figure 4. Characterization of expression-correlated CpGs.

a) Line graph showing the fraction of positive (red) and negative (blue) expression-correlated CpG sites of the MethCORR matrix that are located on the same chromosome as the associated gene. **b)** Graph showing the average DNA methylation standard deviation for bins of MethCORR CpG sites (included in the MethCORR matrix) and non-MethCORR CpG sites in 70 tissue samples of 17 different organ origins (GSE50192[11]; dark and light blue). MethCORR CpG sites are ordered according to their frequency in the MethCORR matrix (high to low) in bins of 1,000 whereas non MethCORR CpGs are divided in bins of 10,000. **c)** Bar chart showing the enrichment results of three independent eFORGE analyses[12] of top expression-correlated CpGs for the cell type-specific genes, *FAP* (blue) *CD3E* (orange) and *EPCAM* (grey). eFORGE identifies overlaps between expression-correlated CpGs and tissue/cell type-specific DNase 1 hypersensitive sites (DHSs) for a predefined panel of tissue types and evaluates if overlaps are greater than expected (i.e. enrichment of cell type specific signal). Here the consolidated DHS profiles of 39 cell types provided by Roadmap Epigenomics Mapping Consortium and negatively expression-correlated CpGs were used as input. Enrichment significance Q-values provided by eFORGE are represented as standard (z-scores) of the $-\log_{10}$(Q-value) to allow visualization of the three independent analyses in one bar chart. Prediction with significant Q-values (<0.01; eFORGE) are indicated by asterisks. **d**) Bar chart showing MCS z-scores across three cell types for the markers *FAP* (blue bars), *CD3E* (orange bars), and *EPCAM* (grey bars). MCS z-score profiles were calculated within a set of public DNA methylation profiles of cell monocultures and tissues (Supplementary Data 13). Only profiles from the relevant myofibroblast cell line IMR90 (GSE41848), purified primary CD3+ T cells (GSE53193), and a CRC cell line (HCC-2998; NCI60) are shown here. **e**) Table showing the five most enriched gene networks identified by analysis of the 50 genes with greatest MethCORR matrix CpG site overlap to *CD3E*, *FAP*, and *EPCAM*, respectively, using the gene list enrichment analysis tool Enrichr[13] and the ARCHSH4 tissue collection. *P*-value and adjusted *P*-values are provided by the Enrichr software.

Supplementary Figure 5

a

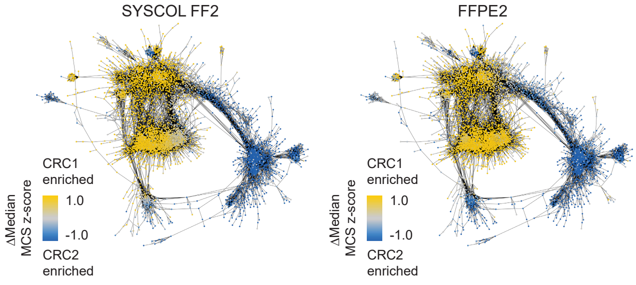

b

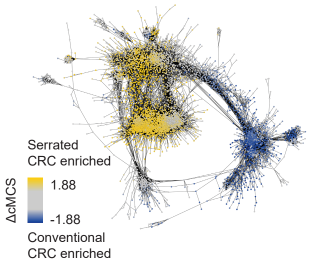

c

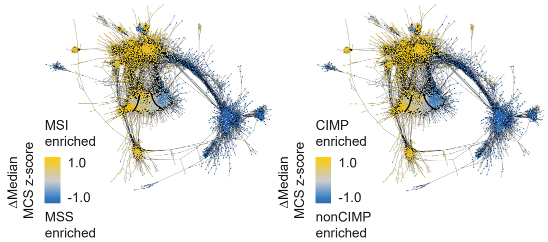

d

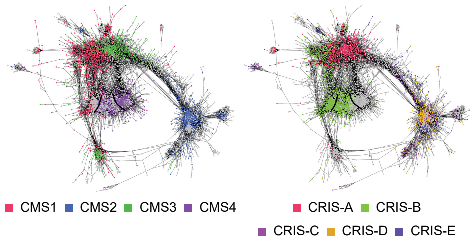

e

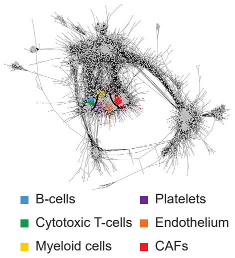

f

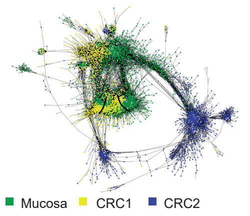

g

Gene set name (Molecular Signature Database v6.2 C2 (curated gene sets)	NES	FDR q-val.	Rank in analysis result
SCHUETZ_BREAST_CANCER_DUCTAL_INVASIVE_UP	3.52	0	1
ANASTASSIOU_MULTICANCER_INVASIVENESS_SIGNATURE	3.49	0	2
VECCHI_GASTRIC_CANCER_ADVANCED_VS_EARLY_UP	3.38	0	3
LIM_MAMMARY_STEM_CELL_UP	3.18	0	4
PICCALUGA_ANGIOIMMUNOBLASTIC_LYMPHOMA_UP	3.17	0	5
LINDGREN_BLADDER_CANCER_CLUSTER_2B	3.07	0	6
BOQUEST_STEM_CELL_UP	3.06	0	7
HOLLERN_EMT_BREAST_TUMOR_UP	2.98	0	8
TURASHVILI_BREAST_LOBULAR_CARCINOMA_VS_DUCTAL_NORMAL_UP	2.98	0	9
NABA_CORE_MATRISOME	2.97	0	10

h

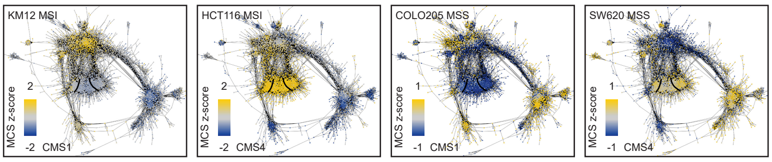

i

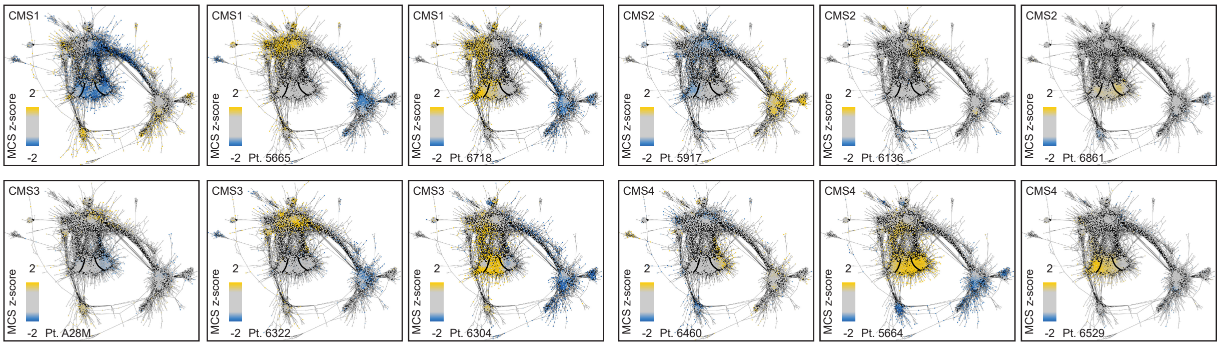

Supplementary Figure 5. A MethCORR map characterizes CRC heterogeneity.

a) MethCORR map with genes colored according to ΔMedian MCS z-scores comparing CRC1 and CRC2 in the SYSCOL FF2 (left) and the FFPE2 (right) cohort. **b)** MethCORR map with genes colored according to their correlation to median MCS difference score (ΔcMCS) calculated for gene-sets defining either serrated (MSigDB M16586) or conventional CRC (MSigDB M17423)[14]. **c)** MethCORR map with genes colored according to ΔMedian MCS z-scores comparing MSI-H and MSS samples (left) or CIMP and non-CIMP samples of the COREAD FF1 cohort (right). MSI and CIMP status annotations were acquired via the UCSC XENA data base[3] phenotype category (MSI: MSI-H vs. MSS from columns CDE_ID_3226963 and MSI_updated_Oct62011; CIMP: COADREAD CIMP c12 vs. COADREAD non-CIMP c11 from column PANCAN DNAMethyl_PANCAN). **d)** MethCORR map with genes colored according to high median MCS z-scores for CMS 1-4 (left) or CRIS A-E subtype samples (right) in the COREAD FF1 cohort. **e)** MethCORR map with genes colored according to high correlation to MCSs of transcriptional biomarkers defining B-cells (*CD79A*) and Myeloid cells (*TREM1*) or high correlation to median MCS (cMCS) calculated for gene sets defining cytotoxic T-cells (MSigDB M13247), platelets (MSigDB M7732), endothelium[2], and CAFs[2]. **f)** MethCORR map with genes colored according to high median MCS z-scores from a comparison of COREAD normal mucosa, COREAD FF1 CRC1 and CRC2 samples. **g)** Table showing the normalized enrichment scores (NESs) from a pre-ranked gene set enrichment analysis[15] (GSEA; MSigDB gene set collection v6.2; C2 curated gene sets) comparing MCSs of 10 CRC2 samples with an epithelial EMT map pattern to 10 CRC2 samples with an early enterocyte pattern from the COREAD FF1 cohort. Top 10 enriched gene sets for the epithelial EMT sample group are shown. **h)** MethCORR maps with genes colored according to the MCS z-scores from the four CRC cell lines KM12, HCT116, COLO205, and SW620. MCS z-scores were calculated using all NCI-60 cell lines and the median MCSs for COREAD FF1 samples. **i)** MethCORR maps with genes colored according to MCS z-scores calculated across the COREAD FF1 cohort of representative CMS1, CMS2, CMS3, and CMS4 samples.

Supplementary Figure 6

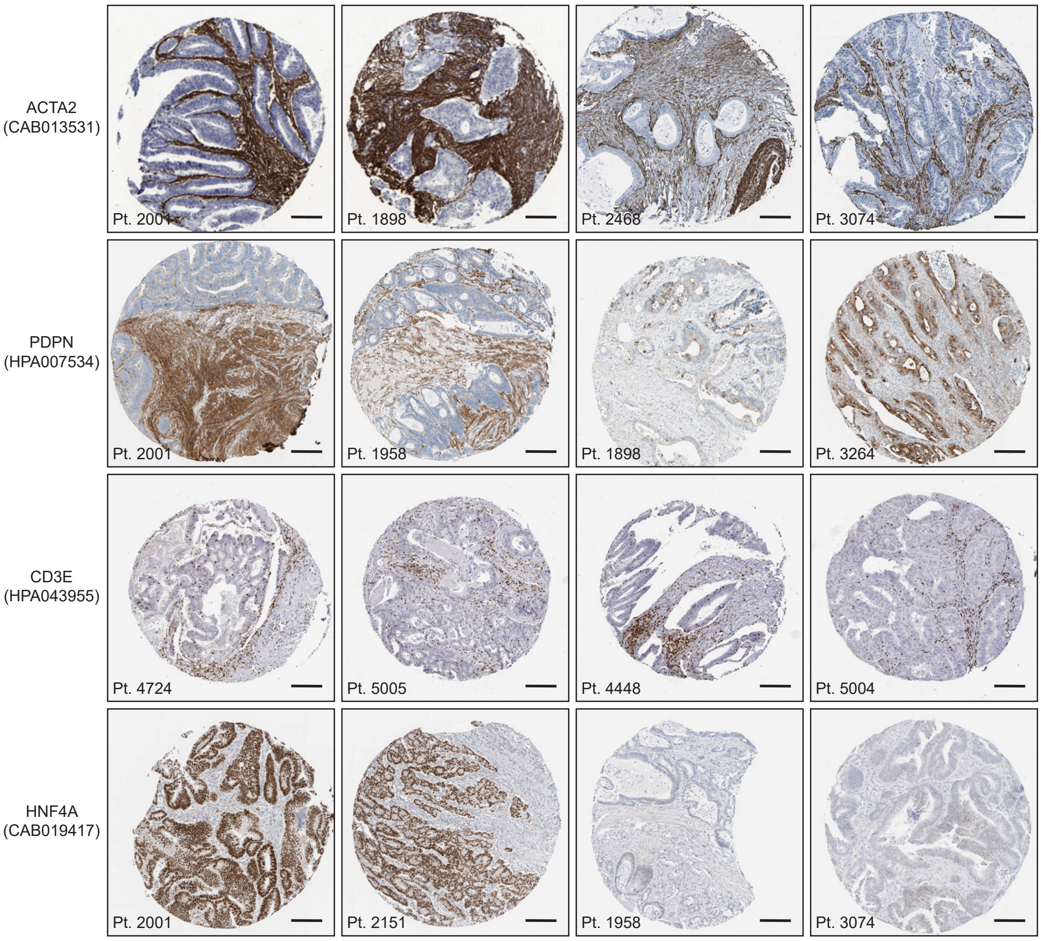

Supplementary Figure 6. Immunohistochemistry profiles of subtype biomarkers.

Immunohistochemistry analysis of representative CRC tissue sections provided by the Human Protein Atlas[16] performed with antibodies specific for ACTA2, PDPN, CD3E, and HNF4A. The following staining patterns were observed: ACTA2 staining was seen with varying intensity within the tumor stroma, which is in agreement with its reported status as a myoepithelial/CAF marker[17]. PDPN staining was identified either within the tumor stroma, in agreement with its reported expression in CAFs, or in cancer epithelial cells in agreement with upregulation in epithelial cells undergoing EMT[18]. CD3E staining was specific to lymphocyte populations within the TME in agreement with its status as a T cell-specific marker[19]. HNF4A staining was specific to cancer epithelial cells, however, pronounced differences in staining between CRC samples was seen in agreement with its potential status as a CRC2 subtype-specific marker. Scale bar: 200 µm.

Supplementary Figure 7

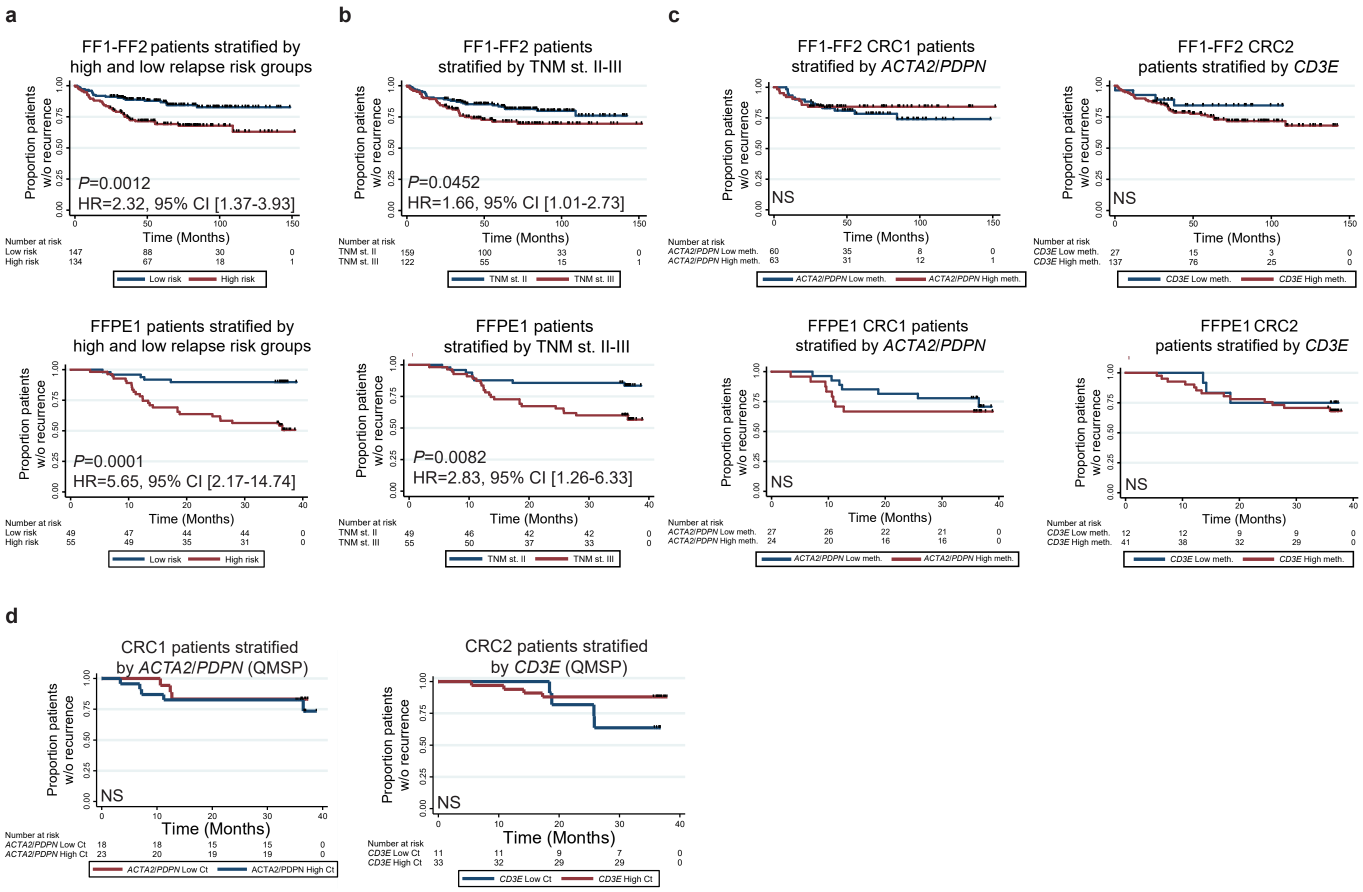

Supplementary Figure 7. Validation of subtype-specific prognostic biomarkers.

a) Kaplan Meier plot showing the relapse-free survival of patients in the FF1+FF2 and FFPE1 cohorts stratified by high and low relapse risk groups. High relapse risk groups are samples with high *CD3E* methylation levels in CRC1 or low average *ACTA2*/*PDPN* methylation levels in CRC2. **b)** Kaplan Meier plot showing the relapse-free survival of patients in the FF1-FF2- and FFPE1 cohorts stratified by TNM stage (III vs. II). **c)** Kaplan Meier plot showing the relapse-free survival of patients stratified by the average CpG methylation level of the *ACTA2*/*PDPN* promoter in CRC1 and the CpG methylation level of the *CD3E* promoter in CRC2 of the combined FF1-FF2- and the FFPE1 cohorts. *P*-values (log-rank test) and HR95%CI are indicated. **d)** Kaplan Meier plot showing the relapse-free survival of CRC1 patients stratified by the *ACTA2*/*PDPN* QMSP assay ΔCt-values and CRC2 patients stratified by the *CD3E* QMSP assay ΔCt-values in a total of 85 FFPE samples from the FFPE1 cohort. *P*-values (log-rank test) and HR95%CI are indicated.

Supplementary Table 1. Demographic, clinical, and pathological overview of patient cohorts used in this study. All samples with matched DNA methylation and RNA expression data from the COREAD and SYSCOL cohorts were used for discovery and validation of the MethCORR method. Primary TNM stage II-III tumor samples with DNA methylation data from the COREAD, SYSCOL, FFPE1, and FFPE2 cohorts were used for subtype discovery. Samples with good clinical annotation and follow up were used for biological characterization and recurrence-free survival analysis (COREAD FF1, SYSCOL FF2, and FFPE1).

	**COREAD**	**SYSCOL**	**COREAD TNM st. II-III**	**SYSCOL TNM st. II-III**	**FFPE1 TNM st. II-III**	**FFPE2 TNM st. II-III**	**COREAD FF1**	**SYSCOL FF2**	**FFPE1**
**Cohort origin**	COREAD TCGA	SYSCOL Denmark	COREAD TCGA	SYSCOL Denmark	COLOFOL Denmark	IDIBELL Spain	COREAD TCGA	SYSCOL Denmark	COLOFOL Denmark
**$N_{subjects}$**	394	314	264	203	113	56	134	153	104
**Sex**									
**Female**	177 (45%)	147 (47%)	128 (48%)	87 (43%)	52 (46%)	9 (16%)	65 (49%)	63 (41%)	48 (46%)
**Male**	213 (54%)	167 (53%)	136 (52%)	116 (57%)	61 (54%)	47 (84%)	69 (51%)	90 (59%)	56 (54%)
**Not available**	4 (1%)								
**Age**									
**<65**	179 (45%)	121 (39%)	119 (45%)	80 (39%)	59 (52%)	24 (43%)	62 (46%)	63 (41%)	51 (49%)
**≥65**	211 (54%)	193 (61%)	145 (55%)	123 (61%)	54 (48%)	25 (45%)	72 (54%)	90 (59%)	53 (51%)
**Not available**	4 (1%)					7 (13%)			
**Tissue type**									
**Colorectal cancer**	373 (95%)	263 (84%)	264 (100%)	203 (100%)	113 (100%)	56 (100%)	134 (100%)	153 (100%)	104 (100%)
**Normal mucosa**	21 (5%)	25 (8%)							
**Adenoma**		26 (8%)							
**Tumor location**									
**Right**	166 (45%)	84 (32%)	121 (46%)	70 (34%)	39 (35%)	27 (48%)	62 (46%)	51 (33%)	36 (35%)
**Left**	102 (27%)	91 (35%)	71 (27%)	71 (35%)	37 (33%)	9 (16%)	39 (29%)	57 (37%)	36 (35%)
**Rectum**	88 (24%)	88 (33%)	62 (23%)	62 (31%)	37 (33%)	20 (36%)	27 (20%)	45 (29%)	32 (31%)
**Not available**	17 (5%)		10 (4%)				6 (4%)		
**TNM stage**									
**TNM st. I**	54 (14%)	42 (16%)							
**TNM st. II**	133 (36%)	116 (44%)	144 (56%)	116 (57%)	54 (48%)	18 (32%)	74 (55%)	88 (58%)	49 (47%)
**TNM st. III**	112 (30%)	87 (33%)	120 (45%)	87 (43%)	59 (52%)	38 (68%)	60 (45%)	65 (42%)	55 (53%)
**TNM st. IV**	51 (14%)	18 (7%)							
**Not available**	23 (6%)								
**MSI/MSS status**									
**MSI**	52 (14%)	48 (18%)	39 (15%)	41 (20%)	20 (18%)	8 (14%)	18 (13%)	33 (22%)	18 (17%)
**MSS**	311 (83%)	213 (81%)	218 (83%)	161 (79%)	93 (82%)	48 (86%)	112 (84%)	119 (78%)	86 (83%)
**Not available**	10 (3%)	2 (1%)	7 (3%)	1 (0.5%)			4 (3%)	1 (1%)	
**Data types**									
**RNA seq.**	RNA seq.	RNA seq.	RNA seq.	RNA seq.	Not available	Not available	RNA seq.	RNA seq.	Not available
**DNA methylation**	450K	450K	450K	450K	EPIC	450K	450K	450K	EPIC

Supplementary Table 2. Table showing R^2, RMSE, and Spearman´s rho for the intra-sample correlation between iRNA, RNA expression, or MCS in FFPE samples and RNA expression or MCS in matched fresh-frozen tissue for nine COREAD samples (related to Figure 1g).

TCGA COREAD patient Id	R^2 iRNA (FFPE) vs RNA (FF)	R^2 RNA (FFPE) vs RNA (FF)	R^2 MCS (FFPE) vs MCS (FF)	RMSE iRNA (FFPE) vs RNA (FF)	RMSE RNA (FFPE) vs RNA (FF)	RMSE MCS (FFPE) vs MCS (FF)	Spearman´s rho iRNA (FFPE) vs RNA (FF)	Spearman´s rho RNA (FFPE) vs RNA (FF)	Spearman´s rho MCS (FFPE) vs MCS (FF)
Pt. 6650	0.94	0.87	1.00	0.47	0.69	0.04	0.97	0.94	1.00
Pt. 5659	0.92	0.74	1.00	0.54	1.08	0.03	0.96	0.87	0.99
Pt. 5661	0.92	0.67	0.99	0.54	1.25	0.03	0.96	0.83	0.99
Pt. 5665	0.91	0.72	0.98	0.57	1.02	0.04	0.95	0.85	0.98
Pt. 6781	0.91	0.69	0.98	0.54	1.00	0.03	0.95	0.82	0.97
Pt. 6780	0.90	0.81	0.99	0.60	0.82	0.03	0.95	0.91	0.99
Pt. 2684	0.88	0.67	0.98	0.65	1.03	0.04	0.94	0.82	0.98
Pt. 3810	0.87	0.70	1.00	0.66	0.98	0.02	0.93	0.83	1.00
Pt. 5656	0.80	0.63	0.98	0.83	1.11	0.07	0.90	0.79	0.98

Supplementary Table 3. Table showing the label and origin of custom gene sets used for pre-ranked Gene Set Enrichment Analysis (GSEA[15]; related to Figure 2d).

Gene set label	Reference
CAFs	[2]
Endothelium	[2]
EMT	[20]
T-cells	[21]
B-cells	[22]
Macrophages	[23]
Dendritic cell	[21]
Myeloid derived suppressor cells	[21]
Wound healing	[24]
Up in CIMP vs. non-CIMP CRC	[25]
Up in non-CIMP vs. CIMP CRC	[25]
Up in MSI vs. MSS CRC	[26]
Up in MSS vs. MSI CRC	[26]
Up in BRAF mut. vs. non-BRAF mut. CRC	[27]
Up in non-BRAF mut. vs. BRAF mut. CRC	[27]
Up in serrated vs. conventional CRC	[14]
Up in conventional vs. serrated CRC	[14]
Genes upreg. by APC	[28]
Genes downreg. by APC	[28]
Up in WNT pathway	[28]
Up in colon crypt top vs. bottom	[29]
Up in colon crypt bottom vs. top	[29]
Undifferentiated cancer	[30]

Supplementary Table 4. Table showing the label and origin of DNA methylation profiles used for interpretation of the MethCORR map (related to Figure 3e).

DNA methylation profile name	Reference
CD3+ T-cells	GSE53193[31]
CD19+ B-cells	GSE35069[32]
CD14+ monocytes	GSE43976[33]
Endothelium	GSE34486[34]
stem cells (embryonic)	GSE31848[35]
CAFs	GSE68851[36]

Supplementary Table 5. DNA sequences for Quantitative Methylation Specific PCR (QMSP) primers.

Gene	Forward primer 5' to 3'	Reverse primer 5' to 3'	Probe 5' to 3'
HNF4A	GGATTTTAGGTTTGGTTAGGTTG	CAATAAATACAACCAACTACCACCA	FAM-TTTTAGTTGTGGGTTTTTTAAGTGATTGGT-BHQ-1
CD3E	TAGAAGTAGTAAGTTTGTTGGTTTTT	CAAATAAAACTATAAACCTTCCAACA	FAM-CTCTCCAATAAATACCCAACTACATCTTT-BHQ-1
ACTA2	ATTTTTTAATTTGGGTGGTTG	CCCACAAACAACTAAACCA	FAM-TGTATTTTATTGGTTTGTTTATGAAATGGG-BHQ1
PDPN	GGTGGTTTTTGTAGTTGTG	CCCTCAAACTCTATTTAACCAACA	FAM-TCTCAAACCAAATTTAAAATTACAAAACCA-BHQ-1
ALUC4	GGTTAGGTATAGTGGTTTATATTTGTAATTTTAGTA	ATTAACTAAACTAATCTTAAACTCCTAACCTCA	FAM-CCTACCTTAACCTCCC-MGB-NFQ

Supplementary References

1 Eisenberg, E. & Levanon, E. Y. Human housekeeping genes, revisited. *Trends Genet* **29**, 569-574, doi:10.1016/j.tig.2013.05.010 (2013).

2 Isella, C. *et al.* Stromal contribution to the colorectal cancer transcriptome. *Nat Genet* **47**, 312-319, doi:10.1038/ng.3224 (2015).

3 Goldman, M., Craft, B., Brooks, A.N., Zhu, J., Haussler, D. The UCSC Xena Platform for cancer genomics data visualization and interpretation. *bioRxiv*, doi:10.1101/326470 (2018).

4 Grossman, R. L. *et al.* Toward a Shared Vision for Cancer Genomic Data. *N Engl J Med* **375**, 1109-1112, doi:10.1056/NEJMp1607591 (2016).

5 Isella, C. *et al.* Selective analysis of cancer-cell intrinsic transcriptional traits defines novel clinically relevant subtypes of colorectal cancer. *Nat Commun* **8**, 15107, doi:10.1038/ncomms15107 (2017).

6 Morris, T. J. *et al.* ChAMP: 450k Chip Analysis Methylation Pipeline. *Bioinformatics (Oxford, England)* **30**, 428-430, doi:10.1093/bioinformatics/btt684 (2014).

7 Gaujoux, R. & Seoighe, C. A flexible R package for nonnegative matrix factorization. *Bmc Bioinformatics* **11**, 367, doi:10.1186/1471-2105-11-367 (2010).

8 Conesa-Zamora, P. *et al.* Methylome profiling reveals functions and genes which are differentially methylated in serrated compared to conventional colorectal carcinoma. *Clin Epigenetics* **7**, 101, doi:10.1186/s13148-015-0128-7 (2015).

9 Hoshida, Y., Brunet, J. P., Tamayo, P., Golub, T. R. & Mesirov, J. P. Subclass mapping: identifying common subtypes in independent disease data sets. *PloS one* **2**, e1195, doi:10.1371/journal.pone.0001195 (2007).

10 Yoshihara, K. *et al.* Inferring tumour purity and stromal and immune cell admixture from expression data. *Nat Commun* **4**, 2612, doi:10.1038/ncomms3612 (2013).

11 Lokk, K. *et al.* DNA methylome profiling of human tissues identifies global and tissue-specific methylation patterns. *Genome Biol* **15**, r54, doi:10.1186/gb-2014-15-4-r54 (2014).

12 Breeze, C. E. *et al.* eFORGE: A Tool for Identifying Cell Type-Specific Signal in Epigenomic Data. *Cell Rep* **17**, 2137-2150, doi:10.1016/j.celrep.2016.10.059 (2016).

13 Chen, E. Y. *et al.* Enrichr: interactive and collaborative HTML5 gene list enrichment analysis tool. *Bmc Bioinformatics* **14**, 128, doi:10.1186/1471-2105-14-128 (2013).

14 Laiho, P. *et al.* Serrated carcinomas form a subclass of colorectal cancer with distinct molecular basis. *Oncogene* **26**, 312-320, doi:10.1038/sj.onc.1209778 (2007).

15 Subramanian, A. *et al.* Gene set enrichment analysis: a knowledge-based approach for interpreting genome-wide expression profiles. *Proceedings of the National Academy of Sciences of the United States of America* **102**, 15545-15550, doi:10.1073/pnas.0506580102 (2005).

16 Uhlen, M. *et al.* Proteomics. Tissue-based map of the human proteome. *Science* **347**, 1260419, doi:10.1126/science.1260419 (2015).

17 Togo, S., Polanska, U. M., Horimoto, Y. & Orimo, A. Carcinoma-associated fibroblasts are a promising therapeutic target. *Cancers (Basel)* **5**, 149-169, doi:10.3390/cancers5010149 (2013).

18 Astarita, J. L., Acton, S. E. & Turley, S. J. Podoplanin: emerging functions in development, the immune system, and cancer. *Front Immunol* **3**, 283, doi:10.3389/fimmu.2012.00283 (2012).

19 Kwak, Y. *et al.* Immunoscore encompassing CD3+ and CD8+ T cell densities in distant metastasis is a robust prognostic marker for advanced colorectal cancer. *Oncotarget* **7**, 81778-81790, doi:10.18632/oncotarget.13207 (2016).

20 Anastassiou, D. *et al.* Human cancer cells express Slug-based epithelial-mesenchymal transition gene expression signature obtained in vivo. *BMC cancer* **11**, 529, doi:10.1186/1471-2407-11-529 (2011).

21 Angelova, M. *et al.* Characterization of the immunophenotypes and antigenomes of colorectal cancers reveals distinct tumor escape mechanisms and novel targets for immunotherapy. *Genome Biol* **16**, 64, doi:10.1186/s13059-015-0620-6 (2015).

22 Palmer, C., Diehn, M., Alizadeh, A. A. & Brown, P. O. Cell-type specific gene expression profiles of leukocytes in human peripheral blood. *BMC Genomics* **7**, 115, doi:10.1186/1471-2164-7-115 (2006).
23 Charoentong, P. *et al.* Pan-cancer Immunogenomic Analyses Reveal Genotype-Immunophenotype Relationships and Predictors of Response to Checkpoint Blockade. *Cell Rep* **18**, 248-262, doi:10.1016/j.celrep.2016.12.019 (2017).
24 Galko, M. J. & Krasnow, M. A. Cellular and genetic analysis of wound healing in Drosophila larvae. *Plos Biol* **2**, E239, doi:10.1371/journal.pbio.0020239 (2004).
25 Ferracin, M. *et al.* The methylator phenotype in microsatellite stable colorectal cancers is characterized by a distinct gene expression profile. *J Pathol* **214**, 594-602, doi:10.1002/path.2318 (2008).
26 Watanabe, T. *et al.* Distal colorectal cancers with microsatellite instability (MSI) display distinct gene expression profiles that are different from proximal MSI cancers. *Cancer Res* **66**, 9804-9808, doi:10.1158/0008-5472.CAN-06-1163 (2006).
27 Popovici, V. *et al.* Identification of a poor-prognosis BRAF-mutant-like population of patients with colon cancer. *Journal of clinical oncology : official journal of the American Society of Clinical Oncology* **30**, 1288-1295, doi:10.1200/JCO.2011.39.5814 (2012).
28 Sansom, O. J. *et al.* Myc deletion rescues Apc deficiency in the small intestine. *Nature* **446**, 676-679, doi:10.1038/nature05674 (2007).
29 Kosinski, C. *et al.* Gene expression patterns of human colon tops and basal crypts and BMP antagonists as intestinal stem cell niche factors. *Proceedings of the National Academy of Sciences of the United States of America* **104**, 15418-15423, doi:10.1073/pnas.0707210104 (2007).
30 Rhodes, D. R. *et al.* Large-scale meta-analysis of cancer microarray data identifies common transcriptional profiles of neoplastic transformation and progression. *Proceedings of the National Academy of Sciences of the United States of America* **101**, 9309-9314, doi:10.1073/pnas.0401994101 (2004).
31 Guillemin, C. *et al.* DNA methylation signature of childhood chronic physical aggression in T cells of both men and women. *PloS one* **9**, e86822, doi:10.1371/journal.pone.0086822 (2014).
32 Reinius, L. E. *et al.* Differential DNA methylation in purified human blood cells: implications for cell lineage and studies on disease susceptibility. *PloS one* **7**, e41361, doi:10.1371/journal.pone.0041361 (2012).
33 Marabita, F. *et al.* An evaluation of analysis pipelines for DNA methylation profiling using the Illumina HumanMethylation450 BeadChip platform. *Epigenetics* **8**, 333-346, doi:10.4161/epi.24008 (2013).
34 Bronneke, S. *et al.* DNA methylation regulates lineage-specifying genes in primary lymphatic and blood endothelial cells. *Angiogenesis* **15**, 317-329, doi:10.1007/s10456-012-9264-2 (2012).
35 Nazor, K. L. *et al.* Recurrent variations in DNA methylation in human pluripotent stem cells and their differentiated derivatives. *Cell Stem Cell* **10**, 620-634, doi:10.1016/j.stem.2012.02.013 (2012).
36 Vizoso, M. *et al.* Aberrant DNA methylation in non-small cell lung cancer-associated fibroblasts. *Carcinogenesis* **36**, 1453-1463, doi:10.1093/carcin/bgv146 (2015).
