# Supplementary material for: MethCORR modelling of methylomes from formalin-fixed paraffin-embedded tissue enables characterization and prognostication of colorectal cancer
Source: Nat Commun. 2020 Apr 24;11:2025. doi: 10.1038/s41467-020-16000-6 (PMC7181739; doi:10.1038/s41467-020-16000-6)
Supplement: Supplementary file 3 — Description of Additional Supplementary Files [file 41467_2020_16000_MOESM3_ESM.pdf]

Description of Additional Supplementary Files

File Name: Supplementary Data 1
Description: TCGA COREAD samples used for development of the UCSC XENA TCGA COREAD MethCORR matrix and regression models.

File Name: Supplementary Data 2
Description: Inter-sample model fit and regression coefficients for the UCSC XENA TCGA COREAD cohort.

File Name: Supplementary Data 3
Description: Top RNA expression-correlated CpGs and MethCORR matrix for the UCSC XENA TCGA COREAD cohort.

File Name: Supplementary Data 4
Description: Intra-sample model fit for UCSC XENA TCGA COREAD samples using the COREAD- and SYSCOL-derived regression models.

File Name: Supplementary Data 5
Description: Intra-sample model fit for SYSCOL samples using COREAD- and SYSCOL-derived regression models.

File Name: Supplementary Data 6
Description: SYSCOL samples used for development of the SYSCOL MethCORR matrix and regression models.

File Name: Supplementary Data 7
Description: Inter-sample model fit for the SYSCOL cohort.

File Name: Supplementary Data 8
Description: TCGA COREAD samples used for development of the NCI GDC TCGA COREAD MethCORR matrix and regression models.

File Name: Supplementary Data 9
Description: Inter-sample model fit for the NCI GDC TCGA COREAD cohort.

File Name: Supplementary Data 10
Description: Intra-sample model fit for the NCI GDC TCGA COREAD cohort.

File Name: Supplementary Data 11
Description: Sample IDs for nine COREAD patients with RNA-sequencing and DNA methylation data from matched fresh-frozen and FFPE tissues.

File Name: Supplementary Data 12
Description: UCSC XENA COREAD and SYSCOL TNM stage II-III patients used for NMF-based consensus clustering.

File Name: Supplementary Data 13

Description: DNA methylation datasets (450K/EPIC) used for identification of cell type-specific methylation.
