# Supplementary material for: MethCORR modelling of methylomes from formalin-fixed paraffin-embedded tissue enables characterization and prognostication of colorectal cancer
Source: Nat Commun. 2020 Apr 24;11:2025. doi: 10.1038/s41467-020-16000-6 (PMC7181739; doi:10.1038/s41467-020-16000-6)
Supplement: Supplementary file 17 — Reporting Summary [file 41467_2020_16000_MOESM17_ESM.pdf]

nature research

Corresponding author(s): Claus Lindbjerg Andersen
Jesper Bertram Bramsen

Last updated by author(s): Mar 18, 2020

Reporting Summary

Nature Research wishes to improve the reproducibility of the work that we publish. This form provides structure for consistency and transparency in reporting. For further information on Nature Research policies, see Authors & Referees and the Editorial Policy Checklist .

Statistics

For all statistical analyses, confirm that the following items are present in the figure legend, table legend, main text, or Methods section.

n/a	Confirmed	
☐	☒	The exact sample size (*n*) for each experimental group/condition, given as a discrete number and unit of measurement
☐	☒	A statement on whether measurements were taken from distinct samples or whether the same sample was measured repeatedly
☐	☒	The statistical test(s) used AND whether they are one- or two-sided<br>*Only common tests should be described solely by name; describe more complex techniques in the Methods section.*
☐	☒	A description of all covariates tested
☐	☒	A description of any assumptions or corrections, such as tests of normality and adjustment for multiple comparisons
☐	☒	A full description of the statistical parameters including central tendency (e.g. means) or other basic estimates (e.g. regression coefficient) AND variation (e.g. standard deviation) or associated estimates of uncertainty (e.g. confidence intervals)
☐	☒	For null hypothesis testing, the test statistic (e.g. *F*, *t*, *r*) with confidence intervals, effect sizes, degrees of freedom and *P* value noted<br>*Give P values as exact values whenever suitable.*
☒	☐	For Bayesian analysis, information on the choice of priors and Markov chain Monte Carlo settings
☐	☒	For hierarchical and complex designs, identification of the appropriate level for tests and full reporting of outcomes
☐	☒	Estimates of effect sizes (e.g. Cohen's *d*, Pearson's *r*), indicating how they were calculated

Our web collection on statistics for biologists contains articles on many of the points above.

Software and code

Policy information about availability of computer code

Data collection	The DNA methylation and RNA sequencing data sets from the CRC patient cohort TCGA COREAD were aquired via public databases whereas datasets from the CRC patient cohort SYSCOL were established by the authors by collection of RNA sequencing and DNA methylation profiling of tissue biopsies taken from Danish individuals with CRC. The generated SYSCOL data sets are deposited at EGA for controlled access according to Danish law, as described in the section "data availability" in the manuscript.
Data analysis	Processing of 450K/EPIC BeadChipMethylation raw data: β-values for each CpG site were derived using the publicly-available ChAMP R-package using the champ.import and champ.norm functions. Missing β-values were imputed using the R-package Impute.<br><br>Processing of RNA sequencing raw data: sequencing reads were mapped to the human genome issue HG19 (hg19) using the publicly available software Tophat2 mapper (Tophat: v2.0.10) and estimating fragments per kilobase of exon per million fragments mapped (FPKM) values for Ensembl genes using the publicly available software Cufflink (Cufflinks: v2.2.1; Gencode v15 annotation w/o Pseudogenes).<br><br>Correlations (Spearman) between RNA expression and DNA methylation were calculated using the publicly available R function "cor".<br><br>Calculations of MethCORR methylation scores (MCSs) from DNA methylation was performed using the formula provided in Figure 1b and the MethCORR Matrix provided in Supplementary Data 3.<br><br>Modelling of RNA expression from MethCORR methylation scores (MCSs): The publicly available Caret R-package was used to perform linear regression modeling by 10x10 fold cross validation and provide R2, RMSE, and MAE measures.<br><br>Correlations (Pearson´s, Spearman´s, and R2) between measured RNA expression and inferred RNA expression were calculated using the publicly available Caret R-package or R function "cor".

Establishment of the MethCORR map: clustering MethCORR genes according to their overlap in expression-correlated CpGs using the publicly available software Cytoscape V3.2.0 and publicly available application EnrichmentMap (Jaccard+Overlap filtering cutoff 0.126).

NMF: Non-negative matrix factorization (NMF) consensus clustering was performed using the publicly available R-package NMF. The similarity of independent subtype predictions was analyzed using the publicly available Genepattern SubMap module (v3).

CMS/CRIS classification: Consensus molecular subtype (CMS) classification was performed with the publicly available R-package CMSclassifier using the single sample method and nearest CMS as predicted subtype. CRC intrinsic subtype (CRIS) classification was performed using the publicly available R package CRISclassifier.

CIN scores: CIN scores were derived from copy number data extracted from the HM-450K/EPIC methylome BeadChips using the champ.CNA module of the publicly available ChAMP R-package.

Stroma/Immune scores were calculated using the publicly available R-package ESTIMATE using default parameters.

GSEA was performed using the publicly available GSEA 3.0 tool using default settings.

Gene list enrichment analysis was performed using the publicly available Enrichr software.

eFORGE analysis was performed using the the publicly available eFORGE software.

Survival analysis was performed using the Kaplan–Meier method with the Stata/IC 14.2 (StataCorp) software.

For manuscripts utilizing custom algorithms or software that are central to the research but not yet described in published literature, software must be made available to editors/reviewers. We strongly encourage code deposition in a community repository (e.g. GitHub). See the Nature Research guidelines for submitting code & software for further information.

Data

Policy information about availability of data

All manuscripts must include a data availability statement. This statement should provide the following information, where applicable:

- Accession codes, unique identifiers, or web links for publicly available datasets
- A list of figures that have associated raw data
- A description of any restrictions on data availability

Normalized 450K DNA methylation datasets for the TCGA COREAD cohort used in this study are publicly available via the UCSC XENA Public Data Hubs [https://tcga.xenahubs.net] using the “dataset ID: TCGA.COADREAD.sampleMap/HumanMethylation450” and via the GDC Data Portal [https://portal.gdc.cancer.gov/] as “datatype=methylation beta value” and “platform=”illumina human methylation 450" for the TCGA-COAD and TCGA-READ project. Normalized RNA sequencing data sets for the TCGA COREAD cohort used in this study are publicly available via the UCSC XENA Public Data Hubs [https://tcga.xenahubs.net] using the “dataset ID: TCGA.COADREAD.sampleMap/HiSeqV2” and via the GDC Data Portal [https://portal.gdc.cancer.gov/] as “Experimental strategy=RNA-Seq” and Workflow Type= HTSeq = FPKM” for the TCGA-COAD and TCGA-READ project. 450K DNA methylation and RNA sequencing data from TCGA CRC patient with matched fresh-frozen and FFPE samples are publicly avialable via the GDC Data Portal [https://portal.gdc.cancer.gov/] using the Database UUID provided in Supplementary Data 11. The RNA sequencing data from the SYSCOL adenoma/carcinoma samples and the SYSCOL 450K methylome data is deposited at European Genome-phenome Archive (EGA, [https://www.ebi.ac.uk/ega/]), which is hosted by the European Bioinformatics Institute (EBI) and the Centre for Genomic Regulation (CRG). Study accession numbers are: EGAS00001002376 (RNA sequencing) and EGAS00001004293 (methylomes). The dataset and sample ID’s of the other publicly available DNA methylation datasets used in this study are given in Supplementary Data 13. All other data supporting the findings of this study are available within the article, its supplementary information files and from the corresponding author upon reasonable request.

Field-specific reporting

Please select the one below that is the best fit for your research. If you are not sure, read the appropriate sections before making your selection.

☒ Life sciences ☐ Behavioural & social sciences ☐ Ecological, evolutionary & environmental sciences

For a reference copy of the document with all sections, see nature.com/documents/nr-reporting-summary-flat.pdf

Life sciences study design

All studies must disclose on these points even when the disclosure is negative.

Sample size	No sample-size calculation was performed.
Data exclusions	In the MethCORR method development and validation phase we did not exclude any samples/data and included all samples with matching RNA sequecning and 450k DNA methylation data. During biological characterization and survival analysis we only used CRC TNM stage II-III samples with good clinical annotation and a minimum of 2-years of follow-up (as these are most relevant for identification of prognostic biomarkers). To avoid confounders in the prognostic analyses we excluded patients diagnosed with synchronous cancers, and patients who were diagnosed with another cancer within 3 years of the CRC diagnosis. Likewise we excluded patients who were diagnosed with local recurrence during follow-up.

Replication	In the MethCORR method development phase we used the publicly avialable normalized data for the TCGA COREAD cohort taken from the UCSC XENA database: the cohort was divided into a discovery part (4/5), which was used for development of the method and a validation part (1/5), which was used for validation of the method.<br>Furthermore, we used the independent SYSCOL cohort (profiled in this study) to replicate/validate the MethCORR method. Finally, we validated our analysis by analyzing the TCGA COREAD dataset provided by the NCI GDC database .<br><br>The existence of the CRC1 and CRC2 molecular CRC subtypes was replicated in independent cohorts. The prognostic value of the DNA methylation-based biomarkers were replicated in independent cohorts using the 450K DNA methylation data and validated using an orthogonal method - Quantitative methylation sensitive PCR (QMSP). QMSP was performed on a subset of random CRC samples for which sample DNA was still available.
Randomization	The division of the sample sets used for development of the MethCORR method (into discovery and validation subsets), were performed using stratified randomization to ensure a balanced distribution of the following variables: Gender, age, UICC stage, microsattelite instability and recurrence status.
Blinding	The analysts generating the in house data (450K methylation array data and QMSP data) were blinded to group allocation. Data generation was performed to avoid batch effects. We cannot account for the public available data. The NMF clustering analysis was performed unsupervised i.e. blinded for group allocation. The discovery of the prognostic markers were performed unblinded, and the biomarkers were validated in independent patients cohorts.

Reporting for specific materials, systems and methods

We require information from authors about some types of materials, experimental systems and methods used in many studies. Here, indicate whether each material, system or method listed is relevant to your study. If you are not sure if a list item applies to your research, read the appropriate section before selecting a response.

Materials & experimental systems

n/a	Involved in the study	
☐	☒	Antibodies
☐	☒	Eukaryotic cell lines
☒	☐	Palaeontology
☒	☐	Animals and other organisms
☐	☒	Human research participants
☐	☒	Clinical data

Methods

n/a	Involved in the study	
☒	☐	ChIP-seq
☒	☐	Flow cytometry
☒	☐	MRI-based neuroimaging

Antibodies

Antibodies used	Immunohistochemical stainings of CRC tissue sections were acquired from the Human Protein Atlas [https://www.proteinatlas.org/]. The following antibody and tissue sections were chosen (available from v8.proteinatlas.org): ACTA2 (antibody: CAB013531; Pt. 2001, Pt. 1898, Pt. 2468, Pt. 3074), PDPN (antibody: HPA007534; Pt. 2001, Pt. 1958, Pt. 1898, Pt. 3264), CD3E (antibody: HPA043955; Pt. 4724, Pt. 5005, Pt. 4448, Pt. 5004), HNF4A (antibody: CAB019417; Pt. 2001, Pt. 2151, Pt. 1958, Pt. 3074).
Validation	n/a

Eukaryotic cell lines

Policy information about cell lines

Cell line source(s)	450K DNA methylation data for cell lines were downloaded from Marmal-aid, Gene Expression Omnibus (GEO), or Array express.
Authentication	n/a
Mycoplasma contamination	n/a
Commonly misidentified lines (See ICLAC register)	n/a

Human research participants

Policy information about studies involving human research participants

Population characteristics	The analysed samples originate from individuals diagnosed with colorectal neoplasia and are either normal mucosa, adenoma or adenomcarcinoma biopsies.<br>The following covariates were collected and used in the study: gender, age, tumor microsatelitte status, tumor histology, UICC stage, adenoma histology, past and post cancer diagnoses (used for exclusion during prognosis analysis), recurrence status, time to recurrence.
Recruitment	Danish CRC samples were collected at hospitals in the central region of Jutland, Denmark from 1999-2013 as part of the SYSCOL and COLOFOL studies. None of the patients received neoadjuvant therapy. Studies were conducted in accordance with Danish law and is approved by local institutional review boards and ethical committees. The Spanish FFPE2 cohort (IDIBELL) encompasses samples collected at Medical Oncology Service of ICO Badalona-Germans Trias i Pujol Research Institute (IGTP), Spain. All Patients were followed according to national clinical guidelines and written informed consent was obtained from all patients.
Ethics oversight	The study was approved by the Danish National Committee on Heath Research Ethics

Note that full information on the approval of the study protocol must also be provided in the manuscript.

Clinical data

Policy information about clinical studies

All manuscripts should comply with the ICMJEguidelines for publication of clinical research and a completedCONSORT checklist must be included with all submissions.

Clinical trial registration	n/a
Study protocol	n/a
Data collection	n/a
Outcomes	n/a
